# Operating room data management: improving efficiency and safety in a surgical block

**DOI:** 10.1186/1471-2482-13-7

**Published:** 2013-03-11

**Authors:** Vanni Agnoletti, Matteo Buccioli, Emanuele Padovani, Ruggero M Corso, Peter Perger, Emanuele Piraccini, Rebecca Levy Orelli, Stefano Maitan, Davide Dell’Amore, Domenico Garcea, Claudio Vicini, Teresa Maria Montella, Giorgio Gambale

**Affiliations:** 1 Department of Emergency, Anesthesia and Intensive Care Unit, Morgagni-Pierantoni Hospital, Forlì, Italy; 2 Department of Computer Engineering and Informatics, Morgagni-Pierantoni Hospital, Forlì, Italy; 3 Department of Management, University of Bologna, Bologna, Italy; 4 Department of Thoracic Surgery, Morgagni-Pierantoni Hospital, Forlì, Italy; 5 Department of General Surgery, Morgagni-Pierantoni Hospital, Forlì, Italy; 6 Department of Ear Nose and Throat and Cervical Facial Surgery, Morgagni-Pierantoni Hospital, Forlì, Italy; 7 Rizzoli Orthopaedic Institute, Bologna, Italy

**Keywords:** Operating room, Surgical path, Management, Indicators, Outcomes, Efficiency, Safety, Sustainability

## Abstract

**Background:**

European Healthcare Systems are facing a difficult period characterized by increasing costs and spending cuts due to economic problems. There is the urgent need for new tools which sustain Hospitals decision makers work. This project aimed to develop a data recording system of the surgical process of every patient within the operating theatre. The primary goal was to create a practical and easy data processing tool to give hospital managers, anesthesiologists and surgeons the information basis to increase operating theaters efficiency and patient safety.

**Methods:**

The developed data analysis tool is embedded in an Oracle Business Intelligence Environment, which processes data to simple and understandable performance tachometers and tables. The underlying data analysis is based on scientific literature and the projects teams experience with tracked data. The system login is layered and different users have access to different data outputs depending on their professional needs. The system is divided in the tree profile types Manager, Anesthesiologist and Surgeon. Every profile includes subcategories where operators can access more detailed data analyses. The first data output screen shows general information and guides the user towards more detailed data analysis. The data recording system enabled the registration of 14.675 surgical operations performed from 2009 to 2011.

**Results:**

Raw utilization increased from 44% in 2009 to 52% in 2011. The number of high complexity surgical procedures (≥120 minutes) has increased in certain units while decreased in others. The number of unscheduled procedures performed has been reduced (from 25% in 2009 to 14% in 2011) while maintaining the same percentage of surgical procedures. The number of overtime events decreased in 2010 (23%) and in 2011 (21%) compared to 2009 (28%) and the delays expressed in minutes are almost the same (mean 78 min). The direct link found between the complexity of surgical procedures, the number of unscheduled procedures and overtime show a positive impact of the project on OR management. Despite a consistency in the complexity of procedures (19% in 2009 and 21% in 2011), surgical groups have been successful in reducing the number of unscheduled procedures (from 25% in 2009 to 14% in 2011) and overtime (from 28% in 2009 to 21% in 2011).

**Conclusions:**

The developed project gives healthcare managers, anesthesiologists and surgeons useful information to increase surgical theaters efficiency and patient safety. In difficult economic times is possible to develop something that is of some value to the patient and healthcare system too.

## Introduction

The global economic and financial crisis is having crucial impact on European healthcare systems, while the Italian healthcare system is one of the most affected [1]. Many countries are facing controversial debates concerning the limitations of medical services and treatments by the national health care systems because of decreasing health care expenditure resources [2]. According to Fuat S. Oduncu Germany spends 11.6% of its Gross Domestic Product (GDP) on health care, that places it fourth in the world after the USA (17.4%), the Netherlands (12%), and France (11.8%) in healthcare expenditure terms [2], while Italy occupies a mid-table positions among the Organization for Economic Co-operation and Development Countries (OEDC) [3]. The mentioned economic problems coupled with an overall cost explosion within the Italian Healthcare Sector has led the Italian government to reconfigure its fiscal priorities, with particular focus on the reduction of public debt and attempts to streamline National Health Service Costs. In view of this, health managers are under pressure to create and implement increasingly efficient operating tools which also guarantee patient safety [4]. The introduction of innovation is a challenge in almost all organizations, but is particularly complicated in organizations where the change effort must overcome the resistance of professionals. Professionals often have deeply entrenched values that are not necessarily consistent with - and often are in direct opposition - to the goals of the organization’s senior management team. In fact, this dilemma is particularly prevalent in healthcare sector organizations, where there is a considerable body of evidence to suggest that physicians have an agenda that is often in total contrast to that of non-clinical managers [5,6]. The development of tools to increase efficiency and improve performance measurement as well as accountability for results, is on the agenda of many public sector organizations [7]. From an external-use perspective, transparency has become a widespread indicator of “good governance” in many different contexts [8]. Moreover, the collection of information through performance measurement can assist these organizations to move toward an improved allocation of resources through management control systems [9]. Indeed, improvements catalyzed by new models of public management in countries such as the United States, United Kingdom, Australia and New Zealand have received extensive coverage in scientific literature [10]. However, there is evidence in public management literature to suggest that some countries have greater difficulties in successfully implementing such innovation, due to a traditional, often hypertrophic state bureaucracy and an atavistic diffidence of innovation which is culturally viewed as a deviation from the safe area of status quo [11]. Italy is one such country. The Italian healthcare system have undergone an extensive process of decentralization in the 90s, devolving organizational and fiscal responsibility to regions. Today in Italy, each region is responsible for the healthcare needs of their inhabitants and are faced with the challenge of improving the effectiveness of health care spending where containment of public spending in healthcare is an overall declared goal. This latter goal is particularly critical as one third of all regions are facing large financial deficits [1]. Aside from the aforementioned diffidence between professionals and management, rigid regulations for working hours of human resources pose a second challenge in Italy. National contracts for healthcare workers and nursing staff (not to mention doctors) foresee payment for a fixed amount of hours. Any extra hours which do not derive from overtime (hours worked immediately after the official end of a shift) or oncall hours, go unpaid. This lack of flexibility compromises the optimization of human resources. Therefore, unlike in the USA or other countries, in Italy it would be impossible to ask a nurse scheduled for an afternoon shift, to work additional hours in the morning, or to call extra staff to clean operating rooms when scheduled staff are struggling to maintain a rapid turnover time. In Italy this flexibility stems from a lack of financial resources (it is not possible to pay workers more or hire extra temporary staff for a few hours every week) as well as from a probable “lacuna legis”. In 2004, the older town public hospital Morgagni Hospital’s facility merged into the second town public hospital Pierantoni Hospital, creating a new expanded facility called the Morgagni-Pierantoni Hospital. An important aspect of this change was the amalgamation of all operating rooms into a single location, the Operating Room Block (ORB), thus bringing together surgeons with a vast array of specializations. This new shared workplace forced staff into overcoming the previous fragmentation of logistics.

### Rationale

The aim of this project is to render the operating room process efficient and safe for patients in terms of clinical risk management. The operating theatre represents one of the most critical hospital units, both in patient safety and financial terms [12,13]. The team has chosen the topic of operating room management because of an urgent need to deliver high quality care with limited resources and the correct management of operating theaters represents an important step towards achieving this. We wanted a system able to elaborate data in line with literature [14-16] in order to identify each phase of patient flow. This study represents the third phase of the process started in 2011. This phase started in January 2009 and finished in December 2011. The project is called “Surgical Patient Path” (SPP) and comprises DRS and an Operating Room Management System (ORMS). ORMS is a data analysis system that processes, analyzes and charts data tracked by DRS.

## Background

This study was developed in house by the Forlì Local Health Authority (Forlì, Italy) within which the Morgagni-Pierantoni Hospital operates. In 2005, in view of the newly created operating room block, the management team of the Local Health Authority gave mandate to a multidisciplinary working group to critically evaluate the system in place. The working group was chaired and coordinated by the healthcare directorate and included anesthesiologists, surgeons, nurses, and engineers. The purpose was to improve the level of efficiency and patient safety within the new ORB, and to ensure a fair distribution of hospital resources among healthcare professionals. Looking at the system as a whole it was difficult to identify all the steps of the surgical patient path and instruments were needed to ensure transparency in data gathering and interpretation. The research team of the Hospital performed two main experimentation periods from 2006 to 2008. The aim of the first experimentation was to develop a system called ‘data recording system’ (DRS) to render the surgical path transparent and intelligible by tracking timestamps along different stages of the surgical path process. Initially we set out simply to define appropriate timeframes which would be useful in measuring the efficiency of the Operating Room Block (Table 1 column A), in line with scientific literature [14,17]. Personal Digital Assistants (PDA datalogic Model PSC Falcon 4220, Datalogic Blackjet - Table 1.1) were selected as hardware to support data entry activity. The PDA software was entirely developed by hospital engineers. The software consisted of a timer to keep track of the timestamps. Login was required by using operators for access and utilization of the software. Upon login, operators could identify the patient, record times and select appropriate timestamps from a digital list. The results of the first experimentation phase showed that our surgical path tracking approach was generally implementable; although the additional workload for operators was acceptable, there was potential for reducing it. PDA software required re-engineering to adapt it more effectively to ORB requirements. It also became evident that system improvement potential would be higher if the quantity of time tracking stamps was increased and entire tracks were registered without data lacks or interruptions. With the results of the first experimentation in mind, the aims of the second experimentation consisted of tracking the whole 16 surgical path process steps proposed by Rotondi et al. (Table 1 column B) [17] and increasing the quantity and quality (reducing incompleteness in tracking) of data concerning the surgical process. To overcome the data quality problems the hospital research team introduced a series of improvements. The nurse anesthetist was identified as the appropriate operator to track the different surgical path timeframes: a PDA was supplied to every nurse. The PDA software was redesigned to enable a closer alignment of time tracking with the logistic path of the patient. The software now contains a series of predefined, standardized steps and prompts the operator to enter the time of each path step, and to complete a minimum number of steps before enabling the registration of a path. The adapted version of the software was aligned closer to ORB logistics and suggests following time registration steps to the operator. If a step is not registered the path automatically appears as incomplete. PDA usage was extended with the introduction and development of a barcode reading system, enabling the scanning not only of patient bracelets, but also of cards which operators used to access software and register room ingress/exit. The barcode reading system was identified as the simplest and fastest way to gather data using PDA, and led to a reduction in data entry errors.

**Table 1:** First and second trial: timings of the surgical path process

**Timing**	**1st Trial**	**2nd Trial**
	Column A	Column B
1	Ward exit	Ward exit
2	Entrance ORB	Entrance ORB
3		Identification by nurse anesthetist
4		Entrance anesthesia room
5	Start anesthesia	Start anesthesia
6		End anesthesia
7	Entrance OR	Entrance OR
8	Start surgical procedure	Start surgical procedure
9	End surgical procedure	End surgical procedure
10	Exit OR	Exit OR
11		Entrance RR
12		Exit RR
13		Identification by healthcare assistant
14		Transport ICU
15		Exit ORB
16		Ward re-entry

*ORB*: operating room block.

*OR*: operating room.

*RR*: recovery room.

*ICU*: intensive care unit.

## Materials and methods

ORMS can be regarded as practical analysis tool embedded in a Oracle Business Intelligence Environment, which processes data to simple and understandable performance tachometers and tables. The analysis of data is based on Macario [14] and Dexter’s studies on ORB efficiency [18-21] and our experience and analysis of tracked data. Data recorded by DRS is sent immediately via wifi connection to a central hospital server which functions as interim storage. At the end of every week data is sent to the ORMS system where they are processed and added to previous data analyses.

**Figure 1:**
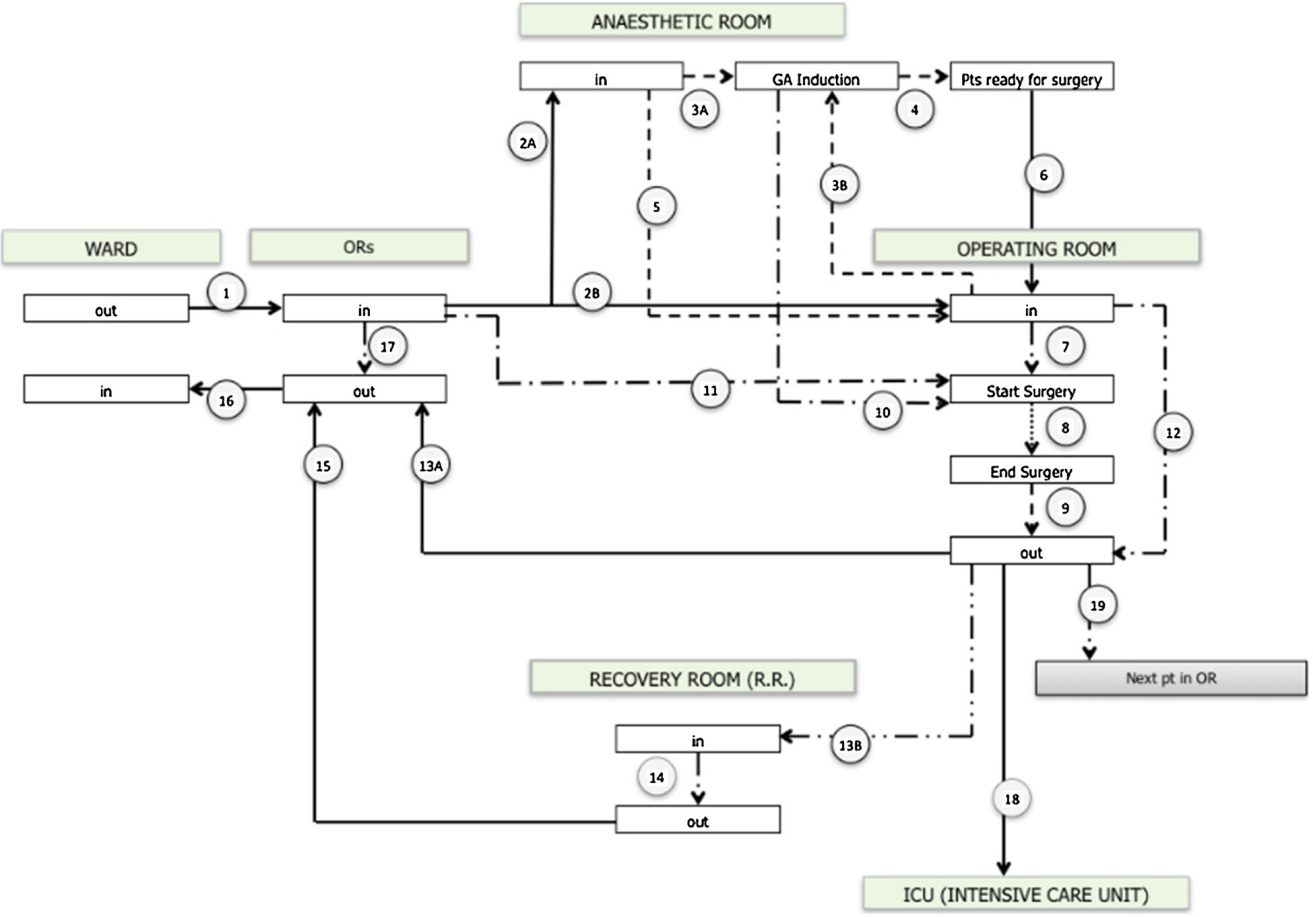
**Surgical path: from the ward to operating room and back to the ward.** Out: patient is leaving. In: patient is entering. ORs: operating rooms or surgical block. GA: general anesthesia. Pt/s: patient/s. R.R.: recovery room. ICU: intensive care unit. A or B: different solution from the same position.

Data is recorded by DRS as a simple output made up of a series of 12 to 16 steps along the pathway from the ward to the operating room. The number of outputs depends on the route the patient follows during the surgical pathway (Figure 1) and data is sent to ORMS as a series of outputs. The system is able to read every step of the surgical path (12–16) and all the delta-times between every step and the next. It is possible to obtain a maximum of 25 delta-times, values which represent a comparison of various times recorded, obtained from the formulae demonstrated in Table 2. Data quality is guaranteed by the introduction of two data quality rules. These data quality rules overcome basic data introduction problems by excluding non reliable data before their analysis. The first rule is that a minimum of 7 path phases are required for a path to be registered. The program automatically defers the registration of a path which fails to contain the minimum number of steps and warns the operator. The second data quality rule excludes unreliable data outliers by introducing minimum and maximum time data input limits for acceptable data values. These limits are defined according to the physician’s indications as results of the first and second trial (Table 3). The ORMS login (with password) is layered and every user has access to data depending on his/her professional needs. The system is divided in tree main profile types (manager), A (anesthesiologist) or S (surgeon); each profile type can access required information in the profile content. Every profile includes a few subcategories where operators can access more detailed data analyses (Table 4). The first data output screen shows general information and guides the user towards more detailed data analysis as precise surgical procedure time of every single surgical units. The hierarchy inside the software enables the user to have a complete insight of data regarding his/her profile in a very simple and clear way. The manager’s profile is aimed at hospital managers and presents data concerning the entity of operations. Within the surgeons profile the business intelligence software works out data which is important for surgeons and anesthesiologists alike.

**Table 2:** Delta times calculated by the timestamps recorded

**n° of delta times and description**	**Formula**
1 Patient moving time from ward to ORB	Entrance ORB – ward exit
2 Waiting time in ORB reception (AR induction)	Identification by NA – entrance ORB
3 Waiting time in ORB reception (OR induction)	Identification by NA – entrance ORB
4 Waiting time for anesthesia in AR	Start anesthesia – entrance AR
5 Waiting time for anesthesia in OR	Start anesthesia – entrance OR
6 Anesthesia time	End anesthesia – start anesthesia
7 Sum of anesthesia time and transport to OR	Identification by NA – entrance in OR
8 Transport time from AR to OR	Entrance in OR – end anesthesia
9 Waiting time in OR	Start surgical procedure – entrance in OR
10 Surgical time	End surgical procedure – start surgical procedure
12 Total pre-surgery time	Exit OR – end surgical procedure
11 Awakening time	Start surgical procedure – identification by NA
13 Total time from admission in ORB	To surgical starting time start surgical procedure – entrance ORB
14 Stay time in OR	Exit OR – entrance OR
15 Waiting time to come back in ward	Exit OR – exit ORB
16 Transport time from OR to RR	Entrance RR – exit OR
17 Stay time in RR	Exit RR – entrance RR
18 Waiting time at reception	Identification by healthcare assistant – exit ORB
19 Transport time to come back in ward	Ward re-entry – exit ORB
20 Stay time in ORB	Exit ORB – entrance ORB
21 Out ORB - transport in ICU	Transport ICU – exit ORB
22 Turnover time	Entrance OR next pts – exit OR previous pts
23 Over-time	Scheduled end of the daily work – exit OR last case
24 Start time tardiness	Scheduled start of the daily work – start surgery 1°case
25 Under utilization	Scheduled end of the daily work – exit OR last case

*ORB*: operating room block.

*AR*: anesthetic room.

*OR*: operating room.

*NA*: nurse anesthetist.

*RR*: recovery room.

*ICU*: intensive care unit.

pts: patients.

**Table 3:** The second data quality rules

**n°**	**Description**	**Limit inferior**	**Limit superior**
1	Patient moving time from ward to ORB	5	20
2	Waiting time in ORB reception (AR induction)	5	20
3	Waiting time in ORB reception (OR induction)	5	20
4	Waiting time for anesthesia in AR	5	20
5	Waiting time for anesthesia in OR	5	20
6	Anesthesia time	10	60
7	Sum of anesthesia time and transport to OR	15	70
8	Transport time from AR to OR	2	10
9	Waiting time in OR	10	60
10	Surgical time	15	720
11	Awakening time	5	30
12	Total pre-surgery time	20	80
13	Total time from admission in OB to surgical starting time	30	120
14	Stay time in OR	20	720
15	Waiting time to come back in ward	5	90
16	Transport time from OR to RR	1	10
17	Stay time in RR	10	180
18	Waiting time at reception	5	20
19	Transport time to come back in ward	5	20
20	Stay time in ORB	40	720
21	Out ORB - transport in ICU	5	20
22	Turnover time	10	120
23	Over-time	30	300
24	Start time tardiness	6	120
25	Under utilization	10	90

*ORB*: operating room block.

*OR*: operating room.

*RR*: recovery room.

*ICU*: intensive care unit.

**Table 4:** Categories and subcategories of data analysis

**Window**	**Subject**	**Level**	**Type of data**
M1	Facility	Global	Quantitative
M2	Productivity units	Comparison	Quantitative
M3	Productivity unit	Comparison	Performance
M4	Facility	Efficient indicators	
M5	Surgical procedure		Qualitative
A1	Facility		Performance
A2	ORB		Pathway
A3	Surgical procedure		Qualitative
A4	Pathway		Timing
S1	Facility	Global	Performance
S2	Productivity unit	Comparison	Quantitative
S3	Surgical procedure		Qualitative
S4	DRG		Quantitative

*M*: Manager.

*A*: Anest+hesiologist.

*S*: Surgeon.

*ORB*: Operating Room Block.

*DRG*: Diagnosis Related Groups.

### Manager (M)

The manager’s profile comprises 5 different data analysis subcategories.

The first output screen (M1) is a global vision of the entire surgical activity in terms of total number of procedures, number of scheduled / unscheduled procedures, raw utilization (total hours of cases performed ÷ total hours of OR time allocated) [22], and a description of all surgical units’ workload.

M2 is a comparison of the productivity of each surgical unit. Variables used to describe the workload are: number of surgical procedures, number of procedures together with duration, and logistic pathway (induction area, ward, recovery room or ICU).

M3 gives a view on surgical units in terms of number of procedures, surgical time average and logistic patient flow analysis (ward, RR or ICU admission).

M4 displays the efficiency indicators and expressed as KPIs (6 dashboards with red, yellow and green color schemes).

M5 represents the Transport-Induction-Surgery-Awakening (TISA) graph. This graph maps the time it takes to bring the patient from the ward to ORB, the induction time, the surgery procedure time and the awakening time. Each time interval is referred to the surgical procedure chosen by the operator, so the TISA graph represents the total amount of time, expressed as average time and standard deviation required to perform a specific procedure.

### Anesthesiologist (A)

The anesthesiologist profile includes 4 different data analysis levels.

A1 shows the total surgical activity in terms of number of anesthesiological procedures and the average anesthesia time (per year and expressed in 12 months).

A2 deals with ORB logistics in term of patient flows. This analysis shows how many patients changed their scheduled pathway and which pathway the patients follow after the surgical procedure (ward, RR, ICU).

A3 displays an Induction and Awakening graph (IA) where anesthesia times are mapped; much like the TISA graph, the average time and the standard deviation is related only to the surgical procedure chosen.

A4 illustrates statistical description (mean, SD, median, min, max) of the all phases of the entire surgical patient pathway. At this level, recorded data is divided into three groups: surgical time, recovery room time and anesthesia time.

### Surgeon (S)

The surgeon profile consists of 4 subdivisions.

S1 represents a general description of the surgical activity. Data displayed includes: the number of procedures, raw utilization, the efficiency indicators and the five most performed surgical procedures (expressed in terms of quantity, average time and standard deviation).

S2 displays a performance comparison between different years/months/weeks. The variables used are: the number of surgical procedures, scheduling analysis (scheduled/unscheduled), logistic patient flow analysis (ward, RR or ICU admission and the number of procedures with a duration of more / less than 120 minutes.

S3 displays an Induction-Surgery-Awakening graph (ISA), similar to the TISA graph, but without the time.

S4 creates a link between the Diagnosis Related Group (DRG) classification and the surgical procedures of a specific surgical unit. The chart presents a quantitative analysis in terms of numbers of surgical procedures per DRG.

## Results

The DRS enabled the registration of 14.675 surgical operations performed over 36 months (from January 2009 to December 2011), and completed data available for ORMS has been gathered for 14.337 patients (97.7%).

The total number of surgical procedures has increased from 4892 in 2009 to 5616 in 2010 and decreased to 5120 in 2011.

The SPP system has improved the efficiency of the operating room process and patient safety.

Raw utilization has increased from 44% in 2009 to 56% in 2010 and decreased to 52% in 2011 with the same OR block time and hours of allocated block time.

The number of high complexity surgical procedures (≥120 minutes) has increased in 2011 compared to 2010 and 2009 for General Surgical unit, ENT surgical unit, Urology surgical unit and Orthopedic-Traumatology surgical units. Thoracic and Vascular surgical units have decreased the percentage from 48 to 45% (Table 5).

**Table 5:** High/Low complexity of surgical procedures

**Surgical unit**		**GS**			**TV**			**ENT**			**UR**			**OT**		
**Years**		**2009**	**2010**	**2011**	**2009**	**2010**	**2011**	**2009**	**2010**	**2011**	**2009**	**2010**	**2011**	**2009**	**2010**	**2011**
High	n^o^	466	486	448	142	191	210	107	127	125	188	186	165	27	80	84
	%	39	42	49	48	48	45	7	9	9	21	22	25	4	6	7
Low	n^o^	730	672	466	154	206	256	1415	1281	1261	706	658	494	639	1254	113
	%	61	58	51	52	52	55	93	91	91	79	78	75	96	94	93

*GS*: General Surgery.

*TV*: Thoracic and Vascular Surgery.

*ENT*: Ear Nose Throat Surgery.

*UR*: Urology Surgery.

*OT*: Orthopedic and Traumatology Surgery.

The number of unscheduled procedures performed has been reduced while maintaining the same percentage of surgical procedures (Table 6).

**Table 6:** Three years analysis of surgical procedures

**Procedures / Years**	**2009**	**2010**	**2011**	**Procedures / Years**	**2009**
Scheduled	75	82	86	Scheduled	75
Unscheduled	25	18	14	Unscheduled	25
High complexity	19	19	21	High complexity	19
Low complexity	81	81	79	Low complexity	81
Over time	28	23	21	Over time	28

Numbers are expressed as %.

High Complexity > 120 minutes.

Low Complexity < 120 minutes.

The number of overtime events decreased in 2010 and in 2011 compared to 2009 and the delays expressed in minutes are almost the same (Table 7).

**Table 7:** Overtime of all surgical units during 3 years

**Overtime**	**2009**	**2010**	**2011**
Number of events	336	371	324
Minutes	78 ± 57*	77 ± 54*	78 ± 54*
Percentage (over surgical procedures)	28	23	21

*Data are means ± SD (range).

A direct link was found between: the complexity of surgical procedures, the number of unscheduled procedures and overtime.

**Figure 2:**
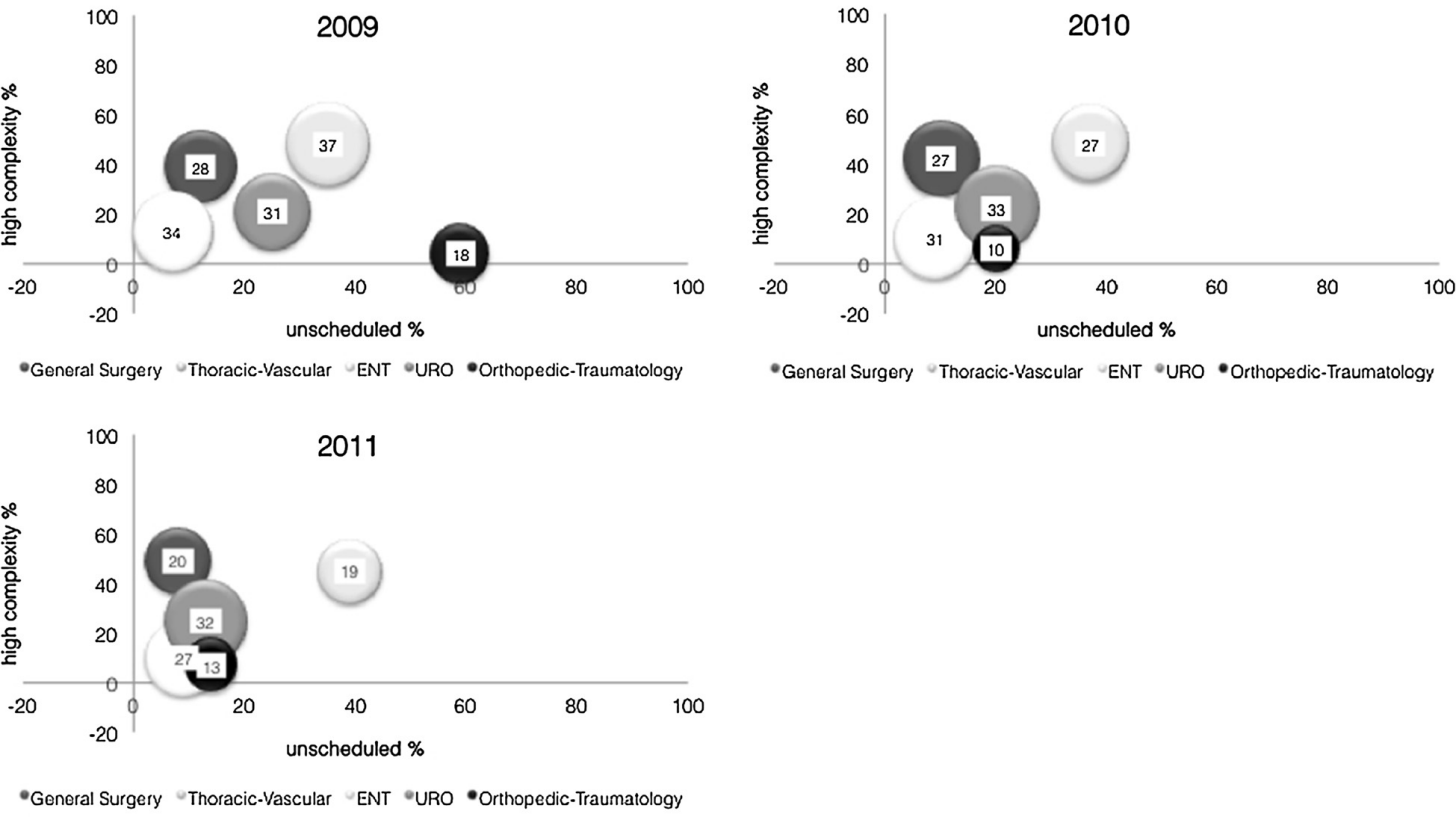
**Three years correlation: complexity - unscheduled procedures - over time.** Surgical Units: General Surgery, Thoracic and Vascular Surgery, Ear Nose and Throat Surgery (ENT), Urology Surgery (URO), Orthopedic and Traumatology Surgery. The bubble diameter stands for percentage of overtime.

Figure 2 shows this link: the X axis represents the percentage of high complexity procedures and the y axis represents the percentage of unscheduled procedures. Bubble diameter represents the percentage of over time procedures.

The graph shows the relation between the three variables; from 2009 to 2011 the bubbles go up or remain at the same height and move closer towards the Y axis as the percentage of unscheduled procedures decreases. Therefore, despite a consistency in the complexity of procedures, surgical groups have been successful in reducing the number of unscheduled procedures and overtime.

No adverse events occurred in three years compared to 24 months (2007–2008), when one event of wrong site surgery (WSS) and 2 near misses of one WSS and of one wrong person surgery (WPS) occurred.

## Discussion

The concept of efficiency has been defined both in terms of cost reduction while maintaining the same level of quality [23], productivity (high throughput, reducing costs and utilizing time properly) and quality [24-28]. This project shows that it is possible to create efficiency and quality starting from a low cost system that is able not only to map each patient’s surgical path every step of the way, but also to provide a clear picture of the complex operating room system on a macro level.

### High throughput

ORMS enables a real-time analysis of the operating room process, and it is capable of elaborating complex data (from inputs to outcomes) not just executing a rudimentary statistical analysis. The number of elaborated outputs depends on the route that patient will follow during the surgical path: a minimum of 12 and a maximum of 16 steps per patient. The total amount of data is derived from 14337 (number of 3 years of surgical procedures) * 12 (172044-outputs). Subsequently 14337*25 delta times produce a total of 358425-outcomes; these figures show just how much data is being analyzed. We are considering changing the second rule cited in materials and methods by adjusting maximum and minimum time limits for each type of surgical procedure. Surgical procedures of different duration would in this way have different predefined ranges.

The workload is considerable, given that it is possible to generate not only annual, monthly or weekly, but even daily reports.

### Low cost project

The set up costs for the SPP (DRS + ORMS) system can be broken down into “start-up costs” and “management costs”. Start-up costs include: 1 PDA for each OR (8) and 2 PDA as replacement back up: a total of10 Personal Digital Assistant (PDA), each costing 1.400€, software and staff training which amounted to 5.000€. The annual costs for the management of the system are 30% of 25000€ = 7500€ (25.000 is the annual salary of the data manager), Software Upgrade 600€ (1 day per year). The real value of SPP can be attributed to its inherent financial sustainability and process sustainability. ORB comprises 8 operating rooms, 3 anesthetic rooms and 1 recovery room. Approximately 5500 surgical procedures are carried out every year and the total cost of the surgical process in ORB is about 6.800.000€ per year. The relationship between the cost of ORMS (13.100€ = 7.500 + 5.000 + 600) and the cost of the total process (6.800.00 0€) is equal to 0.0019% (13.375 ÷ 6.800.000). The annual cost of SPP is 0.0019% of the annual cost of the surgical process: it is a low cost project in the truest sense. Although many professionals involved were able to express their opinion on the project during a series of meetings, no OR Personnel Survey was created or submitted to personnel in order to assess how well OR suites are functioning.

### Reducing costs - utilizing time properly

PDAs, used to gather data in this project, were already being used in all the wards for computed therapy. Only 10 more PDAs were bought. No additional costs were incurred for the development of the project except for the creation of specific software, with particular focus on the development of a user-friendly tool for operators. The PDA's were easy to use and the software was user-friendly; an extensive use of bar code scanning and time stamps to drive the improvement of patient care was achieved. ORMS is based on preexisting knowledge of a re-engineering process; no further equipment was purchased and no additional expenses were accumulated. It was and is a bottom-up project: no money was given by any private company to contribute towards the development of such a system. This method of optimizing existing resources is of particular importance given the current economic climate.

It is important to understand that the increase in the number of surgical procedures in 2010 compared to 2009 was due to an improvement in the raw utilization. In 2011 there was a dip in the total number of surgical procedures attributable to an increase in complexity compared to 2010 and 2009. No additional allocated hours were given by the board to surgical units, a significant achievement considering the reduction in unscheduled procedures.

### Quality

This project can answer these questions: “what can we do for patient safety?” and “how can we improve risk control?” The team initially set out to map the surgical path; today, their research has created a system which not only maps and quantifies but also controls and introduces gates inside the surgical path. SPP can be defined as the main product of this research and it is much like a tree with many branches for patient quality: steps for patient identification (three) and steps to avoid WSS or WPS. PDA also informs doctors and nurses of types of operation, number of operating room, site of surgery (if any), allergies (if any). The right patient is in the right theatre with the right nurses and doctors.

The workgroup is also thinking of inserting all available checklist structures onto PDA's so that a single instrument may be used for several different applications.

This project has many limitations; two surgical units decided not to use the PDAs (Breast and Ophthalmology Units) because of logistic problems (they are located far from the surgical block) and another limit is cultural. Our staff was not ready to share information regarding performance- but the power of data and dialogue are modifying our behavior and today we are aware of our limitations and we are trying to engender greater transparency and safety through the use of data. We realized that the workgroup was not ready to share information because we did not collect any feedback from the operating room team not only during the first part, but also during all successive phases of the project. The workgroup was so focused on the first step that it failed to include all staff members in the theoretical part of the system. The results of this project are producing a "domino effect" not only on surgical or anesthesiological or nursing activities, but also on how we understand the process as a whole. James Harrington [29] states that we can’t improve what we can’t measure; we have improved and strive to improve even further so that our daily work benefits from efficiency, cost reductions and work comprehension.

It is our belief that a secondary effect of our system is the forging of a new way of thinking among team members, a limitation of the project was a failure to collect feedback from operating room teams, not only during the first part, but also during all successive phases of the project. Erebouni et al. write that “different definitions of the concepts of efficiency and productivity in operating departments may lead to confusion among team members”: the power of data and dialogue are modifying our behavior and today we are aware of our limitations and we are trying to engender greater transparency and safety through the use of data. A next step would be to gather data to prove whether members of operating room teams do indeed have a clearer understanding of goals and responsibilities and whether the project engendered an organization-oriented understanding of efficiency.

## Conclusions

This project represents a successful experiment of the introduction of managerial innovation in a public hospital of one such country, Italy. It is interesting to note that although the project was developed by healthcare professionals, it aims to align managerial and professional goals. This is an important step forward, when compared to solutions typically based on a "trade-off" between efficiency (managerial side) and effectiveness (professional side). Further research might be done with the aim to capture which were the contextual enablers of this project and how it could be replicated in other hospitals and countries.

## Abbreviations

A: Anesthesiologist; DRG: Diagnosis Related Group; DRS: Data Recording System; GPD: Gross Domestic Product; IA: Induction-Awakening; ICU: Intensive Care Unit; ISA: Induction-Surgery-Awakening; KPIs: Key Performance Indicators; M: Manager; OEDC: Economic Co-operation and Development Countries; ORB: Operating Room Block; ORMS: Operating Room Management System; PDA: Personal Digital Assistant; RR: Recovery Room; S: Surgeon; SPP: Surgical Patient Path; TISA: Transport-Induction-Surgery-Awakening; USA: United States of America; WSS: Wrong Site Surgery; WPS: Wrong Person Surgery

## Competing interests

The authors declare that they have no competing interests.

## Authors’ contributions

VA: contributed to the planning, data analysis, writing manuscript, interpretation of data, manuscript revision. BM: contributed to the planning, data analysis, writing manuscript, interpretation of data, manuscript revision. EP: contributed to the planning, data analysis, writing manuscript, manuscript revision. RMC: contributed to the planning, data analysis, writing manuscript. PP: contributed to the planning, data analysis, writing manuscript, manuscript revision. EP: contributed to data analysis, writing manuscript, manuscript revision. RLO: contributed to the planning, data analysis, writing manuscript. SM: contributed to data analysis, writing manuscript. DD: contributed to data analysis, writing manuscript. DG: contributed to data analysis, writing manuscript. CV: contributed to the planning, data analysis, writing manuscript. MTM: contributed to data analysis, writing manuscript, manuscript revision. GG: contributed to the planning, data analysis, writing manuscript. All authors read and approved the final manuscript.

## Authors’ information

VA Medical Doctor Specialized in Anesthesiology. BM Biomedical engineer. His particular interest in healthcare management inspired him to create and develop this system. PE Professor of economics. Main area of interest: management. Co-author of several healthcare management text books. CMR Medical Doctor Specialized in Anesthesiology. PP Economics student. PE Medical Doctor Specialized in Anesthesiology. OLR Professor of economics. Main area of interest: management. MS Medical Doctor Specialized in Anesthesiology. DAD Head of Thoracic Department. GD Head of General Surgery Department. VC Professor of ENT Surgery and Head of ENT and Cervical Facial Surgery Department. MMT Hospital Medical Director of Rizzoli Orthopaedic Institute. Expert in healthcare management. GG Head of Emergency Department.

## Pre-publication history

The pre-publication history for this paper can be accessed here:

http://www.biomedcentral.com/1471-2482/13/7/prepub

## References

[B1] Giulio De Belvisa A Ferrè F Specchia ML The financial crisis in Italy: implications for the healthcare sector Health Policy 2012 106 10 16 10.1016/j.healthpol.2012.04.003 22551787

[B2] Oduncu FS Priority-setting, rationing and cost-effectiveness in the German health care system Med Health Care Philos 2012 Published online: Jun 13 10.1007/s11019-012-9423-7 22692518

[B3] OECD (2011): Health at a Glance 2011: OECD Indicators 2011 OECD Publishing http://dx.doi.org/10.1787/health_glance-2011-en

[B4] McKee M Karanikolos M Belcher P Austerity SD A failed experiment on the people of Europe Clinical Medicine 2012 12 346 350 22930881 10.7861/clinmedicine.12-4-346 PMC4952125

[B5] Young D Saltman RB The Hospital Power Equilibrium: Physician Behavior and Cost Control 1985 Baltimore: The Johns Hopkins Press

[B6] Young D Management Accounting in Health Care Organizations 2008 New York: John Wiley & Sons

[B7] Pollitt C Bouckaert G Public Management Reform: A Comparative Analysis-New Public Management, Governance, and the Neo-Weberian State 2011 Oxford: Oxford University Press

[B8] O’Neill O Christopher H, David H Transparency and the ethics of communication Transparency: The Key to Better Governance? 2006 Oxford: Oxford University Press 75 90

[B9] Padovani E Young D Managing Local Governments: Designing Management Control Systems That Deliver Value 2012 Milton Park: Routledge

[B10] Ongaro E Introduction: the reform of public management in France, Greece, Italy, Portugal and Spain The Int J Public Sec Manag 2008 21 101 117 10.1108/09513550810855618

[B11] Dunleavy P Hood C From old public-administration to new public management Public Money Manage 1994 14 9 16

[B12] Guerriero F Guido R Operational research in the management of the operating theatre: a survey Health Care Manag Sci 2011 14 89 114 10.1007/s10729-010-9143-6 21103939

[B13] Maryamaa RA Kirvela OA Who is responsible for operating room management and how do we measure how well we do it? Acta Anaesthesiol Scand 2007 7 809 814 10.1111/j.1399-6576.2007.01368.x 17635390

[B14] Macario A Are Your Hospital Operating Rooms “Efficient”?: A Scoring System with Eight Performance Indicators Anesthesiology 2006 105 237 240 10.1097/00000542-200608000-00004 16871055

[B15] Williams BA DeRiso BM Figallo CM Benchmarking the perioperative process: III. Effects of regional anesthesia clinical pathway techniques on process efficiency and recovery profiles in ambulatory orthopedic surgery J Clin Anesth 1998 7 570 8 9805698 10.1016/s0952-8180(98)00083-x

[B16] McIntosh C Dexter F Epstein R The Impact of Service-Specific Staffing, Case Scheduling, Turnovers, and First-Case Starts on Anesthesia Group and Operating Room Productivity: A Tutorial Using Data from an Australian Hospital Anesth Analg 2006 103 1499 1516 10.1213/01.ane.0000244535.54710.28 17122231

[B17] Rotondi AJ Brindis C Cantees KK Benchmarking the perioperative process. I. Patient routing systems: a method for continual improvement of patient flow and resource utilization J Clin Anesth 1997 2 159 169 9075043 10.1016/s0952-8180(96)00242-5

[B18] Dexter EU Dexter F Masursky D Both bias and lack of knowledge influence organizational focus on first case of the day starts Anesth Analg 2009 108 1257 61 10.1213/ane.0b013e31819a6dd4 19299797

[B19] Wachtel RE Dexter F Influence of the operating room schedule on tardiness from scheduled start times Economics, Education, and Policy 2009 108 1889 1901 10.1213/ane.0b013e31819f9f0c 19448219

[B20] Dexter F Epstein RH Marcon E Estimating the influence of prolonged turnover times and delays by time of day Anesthesiology 2005 102 1242 1248 10.1097/00000542-200506000-00026 15915039

[B21] Wachtel RE Dexter F Reducing tardiness from scheduled start times by making adjustments to the operating room schedule Anesth Analg 2009 108 1902 1909 10.1213/ane.0b013e31819f9fd2 19448220

[B22] Dexter F Traub RD How to schedule elective surgical cases into specific operating rooms to maximize the efficiency of use of operating room time Anesth Analg 2002 94 4 933 42 10.1097/00000539-200204000-00030 11916800

[B23] Arakelian E Gunningberg L Larsson J How operating room efficiency is understood in a surgical team: a qualitative study Int J Qual Healthc 2011 23 100 106 10.1093/intqhc/mzq063 21098628

[B24] Walker R Adam J Changing time in an operating room suite Int J Nurs Stud 2001 38 25 35 10.1016/S0020-7489(00)00057-2 11137720

[B25] Landor P More for Less - But How? Productivity and Efficiency Apprehension in Official and Company Activities 1990 Abo: Abo Akademi

[B26] Ljunggren U An evaluation of methods to measure productivity and efficiency at school—applying in city of Stockholms’ elementary schools 1999 Edsburk: EFI: Akademitryck AB

[B27] Kielhorn A Graf von der Schulenburg JM The Health Economics Handbook 2000 Chester: Aldis International

[B28] Pandit JJ Westbury S Pandit M The concept of surgical operating list ‘efficiency’: a formula to describe the term Anaesthesia 2007 62 895 903 10.1111/j.1365-2044.2007.05174.x 17697215

[B29] Harrington HJ The Improvement Process: How America's Leading Companies Improve Quality 1987 New York: McGraw-Hill Education

